# Visual attention predicts risky supplement decisions in recreational fitness: evidence from a quiet eye experiment

**DOI:** 10.3389/fpsyg.2026.1797048

**Published:** 2026-05-12

**Authors:** René Paasch, Gunnar Mau

**Affiliations:** 1 Department of Health and Sport Sciences, DHGS Deutsche Hochschule für Gesundheit und Sport GmbH, Berlin, Germany; 2 Department of Health Sciences, Hochschule Magdeburg-Stendal, Magdeburg, Germany

**Keywords:** eye-tracking, quiet eye, risk perception, risk acceptance, supplement use, decision-making, visual attention

## Abstract

**Background:**

Risky, high-stimulant supplement use and IPED-like products are increasingly common in recreational fitness environments and represent a relevant public health and harm-reduction concern. While psychosocial correlates of such decisions have been widely examined, less is known about visuocognitive processes involved in the real-time evaluation of supplement-related information at the point of choice. Quiet Eye (QE), defined as the final fixation before an action, has been described as a marker of attentional stability but has not yet been examined in the context of supplement-related risk decision-making.

**Methods:**

A laboratory eye-tracking study was conducted with 166 recreational fitness athletes. Participants viewed a standardized supplement stimulus containing reward-related and warning-related cues, completed a binary task-based risky-choice task, and reported their intention to use the risky product. Measures of QE fixation duration, psychosocial risk constructs, and experimentally induced cognitive load were obtained. Logistic and linear regression models were estimated using standardized predictors.

**Results:**

Longer QE fixation on warning cues was associated with a lower likelihood of selecting the risky supplement (OR = 0.50, *p* = 0.003) and with lower intention scores (*b* = −4.20, *p* < 0.001). In standardized terms, a one-SD increase in QE_warning corresponded to an approximately 50% reduction in the odds of choosing the risky option and a decrease of about four points on the 0–100 intention scale. QE fixation on reward cues was associated with intention but not with task-based choice. Psychosocial variables showed consistent associations with both outcomes: risk acceptance was positively associated with intention and risky choice, whereas risk perception was inversely associated with both. Cognitive load was associated with higher intention scores (*b* = 2.02, *p* = 0.002) but was not associated with task-based choice.

**Conclusion:**

The findings indicate that warning-related QE fixation is associated with lower intention and a reduced likelihood of risky choice in supplement-related decisions. QE metrics are interpreted as visuocognitive markers of attentional allocation during the final phase of decision-making rather than as causal determinants. Together with established psychosocial correlates, visuocognitive attention may constitute an additional descriptive component in understanding supplement-related risk decision processes, with potential relevance for harm-reduction approaches in visually dense fitness environments.

## Introduction

The non-medical use of high-stimulant supplements, hereafter referred to as *risky supplements* due to their IPED-like properties and ambiguous safety profiles, has become increasingly common in recreational fitness environments. Although many athletes perceive these products as benign performance enhancers, prior research indicates that users often underestimate potential health risks while overestimating expected benefits (Sagoe et al., 2014; Kanayama et al., 2020). Decisions regarding such products rarely occur under calm or reflective conditions. Instead, gym environments and digital fitness cultures expose individuals to dense visual information, including performance claims, warning statements, and aesthetic ideals, which must be processed rapidly. How individuals visually engage with these cues may therefore be associated with decision tendencies in supplement-related contexts.

Risky supplements occupy a conceptual position between conventional sports nutrition products and pharmacological image and performance-enhancing drugs (IPEDs). Previous research has described this space as a continuum ranging from benign supplementation to so-called “grey-zone” enhancers and, ultimately, to established doping substances (Backhouse et al., 2007; Petróczi and Aidman, 2009; Christiansen et al., 2023). The products examined in the present study share motivational, perceptual, and risk-related characteristics with IPED use but do not constitute doping in a regulatory sense. Situating these products within a continuum perspective allows the decisions observed in this study to be considered as an early-stage form of enhancement-related risk decision-making.

Although risky supplements are not classified as classical anabolic-androgenic steroids (AAS), prior work in the doping field suggests that athletes may move flexibly across different points of the enhancement continuum depending on perceived risk, performance goals, social norms, and product availability (Backhouse et al., 2013; Petróczi and Aidman, 2009; Christiansen et al., 2023). From this perspective, supplement-related decisions may represent a transitional segment within broader enhancement practices that is relevant for harm-reduction and prevention-oriented research. Empirical work has identified associations between non-medical anabolic-androgenic steroid use and psychological vulnerability factors, underscoring the relevance of examining early decision processes that may precede regular use within recreational fitness contexts (Pope et al., 2012).

Motivational frameworks such as the Theory of Planned Behavior (TPB; Ajzen, 1991, 2015) have been shown to be associated with supplement and doping-related decision-making (Lucidi et al., 2008; Goulet et al., 2010). Meta-analytic evidence indicates that intention and perceived behavioral control are robust correlates of health-related behaviors across domains, providing a well-established descriptive basis for their inclusion in the present study (Armitage and Conner, 2001). Constructs such as attitudes, risk acceptance, risk perception, and perceived task based control are consistently related to intention formation and self-reported behavior. However, these models primarily address motivational dispositions and do not explicitly consider how visual information is processed during the brief moments in which product-related decisions unfold, particularly under conditions of high visual density and time pressure.

A complementary perspective can be found in visuocognitive attention research, particularly the concept of the Quiet Eye (QE). QE is defined as the final, stable fixation on a task-relevant cue prior to action initiation (Vickers, 1996, 2016). In perceptual–motor domains, longer QE durations have been associated with measures of attentional stability and task-focused visual engagement (Vine et al., 2014; Wilson et al., 2019; Causer et al., 2011; Moore et al., 2012). To date, however, QE has not been examined in the context of supplement-related or health-risk decision-making. In the present context, QE is therefore conceptualized as a momentary visuocognitive marker of attentional allocation rather than as a causal mechanism.

This gap has received limited empirical attention. From a descriptive standpoint, visual fixation patterns may differ depending on whether individuals attend primarily to reward-related or warning-related information when evaluating risky supplements. Visual stabilization on reward cues may be related to approach-oriented processing, whereas sustained fixation on warning cues may be associated with increased attention to risk-related information. Within this framing, QE can be understood as complementing motivational constructs derived from TPB-based research by capturing moment-to-moment allocation of visual attention, without implying causal influence. Changes in the immediate decision environment, such as the visual salience of specific information, have been conceptualized as elements of choice architecture that may shape behavior by directing attention at the moment of choice, without presupposing changes in underlying preferences (Hollands et al., 2013).

Situational factors may further shape visual engagement with risk-related information. Cognitive load has been associated with reduced executive control and altered attentional allocation (Lavie, 2005). Given that fitness environments are often characterized by noise, physical fatigue, and high visual stimulation, experimentally induced cognitive load may be relevant when examining how individuals process supplement-related cues under controlled laboratory conditions.

### Present study

The present study examined associations between QE fixation patterns, psychosocial determinants derived from TPB, and cognitive load in risky supplement decision-making among recreational fitness athletes. Using a controlled laboratory eye-tracking paradigm, the study focused on how visual attention to reward-related and warning-related cues relates to intention and binary task-based choice.

Based on prior literature, the study examined the following directional expectations regarding associations:

1. Longer QE fixation on warning cues was expected to be associated with lower intention to use the risky supplement and a lower likelihood of selecting the risky option.
2. Longer QE fixation on reward cues was expected to be associated with higher intention to use the risky supplement.
3. Risk acceptance and risk perception were expected to show consistent associations with both intention and task-based choice.
4. Experimentally induced cognitive load was expected to be associated with higher intention scores.

By combining visuocognitive measures with established psychosocial constructs, this study aims to describe how moment-to-moment visual attention and motivational factors co-occur during risky supplement decision-making in recreational fitness contexts, without making claims regarding causal mechanisms.

## Method

### Design

The study employed a laboratory-based, between-subjects experimental design combining objective eye-tracking, a binary risky-supplement choice task, and validated psychosocial risk measures. Participants completed a cognitive load manipulation (low vs. high), viewed standardized supplement stimuli containing reward and warning cues, and made both task based and intentional decisions regarding product use. This design allowed us to examine visuocognitive markers (Quiet Eye; QE), psychosocial determinants, and situational cognitive load in supplement-related decision-making processes under controlled conditions, with specific relevance for health-oriented and harm-reduction perspectives in recreational fitness settings.

### Participants

A total of 166 recreational fitness athletes were recruited through fitness centers, university sport programs, and online advertisements. Inclusion criteria were: age ≥ 18 years, active engagement in fitness or strength training ≥ 2 times per week, and normal or corrected-to-normal vision.

- Age: M = 28.19 years (SD = 5.94, range 18–50)
- Training years: M = 6.37 (SD = 4.29)
- Sex distribution: 61.4% male, 38.6% female

No missing data occurred for questionnaire variables; eye-tracking data quality was ensured via predefined calibration and exclusion criteria (see Materials and Measures). Participants provided written informed consent and received a small compensation. All procedures complied with the Declaration of Helsinki and were approved by an institutional ethics board. Data were collected in Germany between Septe and November 2025.

### Materials and measures

#### Eye-tracking apparatus

Eye movements were recorded using a 60-Hz infrared eye-tracking system (Tobii) and analyzed using fixation-based metrics. Stimuli were presented on a 15-inch monitor at an approximate viewing distance of 65 cm. Participants were seated in a fixed position and instructed to minimize head movements throughout the task.

**Figure 1:**
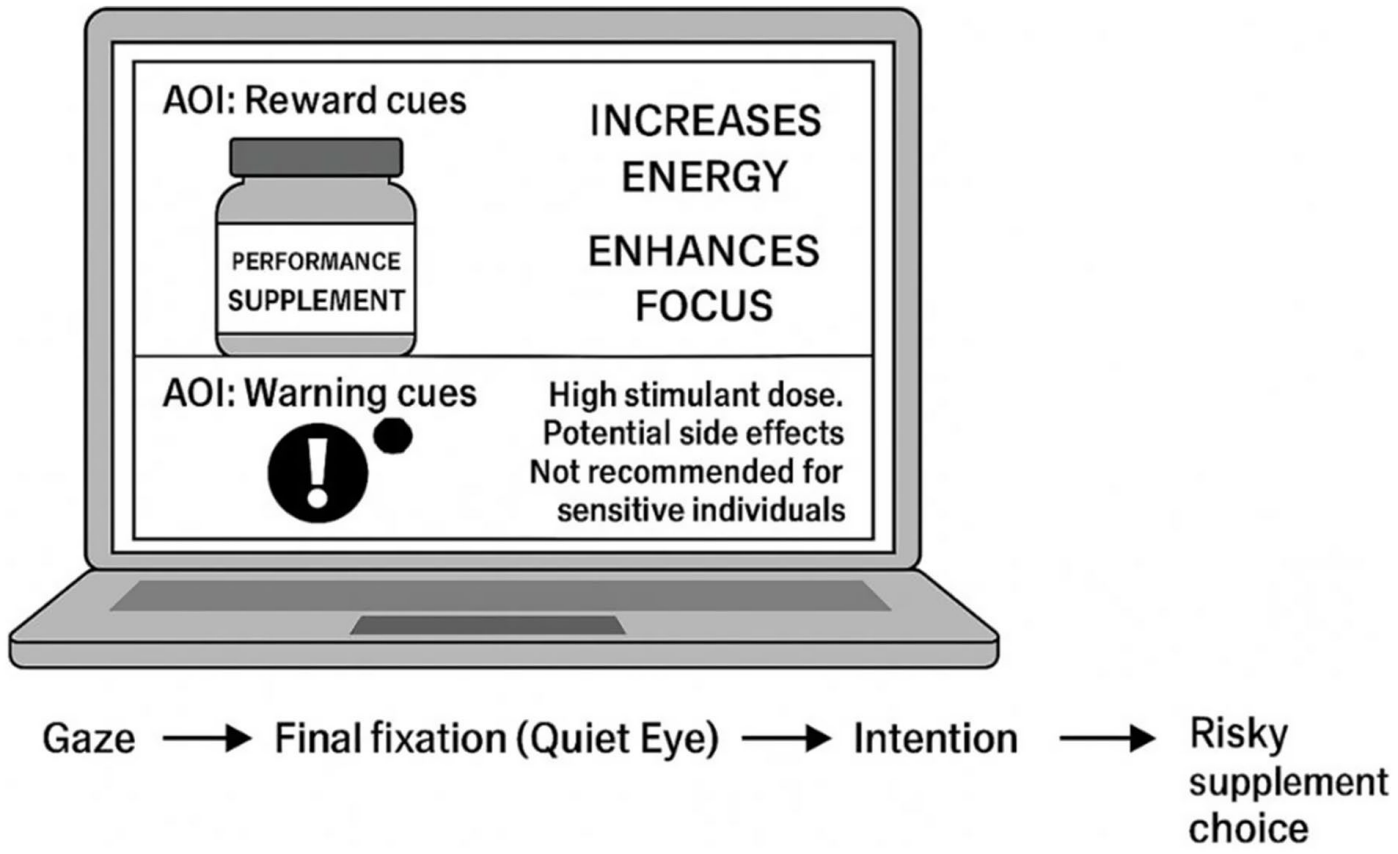
Illustrates the standardized supplement stimulus and the predefined reas of nterest (AOIs) used in the eye-tracking task.

A nine-point calibration procedure was performed immediately prior to the decision task. Calibration was accepted only if the average validation error was ≤ 1° of visual angle. This criterion was applied consistently across all participants to ensure sufficient spatial accuracy for fixation-level analyses of predefined Areas of Interest (AOIs). Given the static stimulus presentation and the exclusive focus on fixation duration rather than saccade dynamics or microsaccadic activity, a 60-Hz sampling rate provides sufficient temporal resolution for reliable fixation detection in the present paradigm Figure 1.

### Stimuli and areas of interest (AOIs)

Participants viewed a standardized supplement image purposely designed for this study. The stimulus contained two clearly defined AOIs:

1. Reward AOI

- Performance claims (“Extreme Energy,” “Max Performance”)
- Bright-colored branding elements
- Designed to reflect approach-related visual information

1. Warning AOI

- Risk statements (“High stimulant dose,” “Potential side effects”)
- Small hazard icon
- Designed to reflect risk-related visual information

AOIs were predefined and applied identically for all participants. AOI boundaries were defined *a priori* based on the fixed stimulus layout and specified in pixel coordinates. The AOIs did not overlap and remained identical across all participants. Boundary placement was reviewed to ensure that reward-related and warning-related visual elements were fully contained within their respective AOIs and that no task-irrelevant stimulus features were included.

Item-level responses were recorded and aggregated into mean composite scores for each construct. Internal consistencies were satisfactory across all scales (risk acceptance *α* = 0.82; risk perception α = 0.80; attitudes *α* = 0.86; perceived behavioral control α = 0.83). Example items include: “I am willing to take health risks if the performance benefits are high” (risk acceptance) and “Using strong performance boosters can cause serious long-term harm” (risk perception). All four-item scales demonstrated clean one-factor structures (primary loadings > 0.60). Item-level responses were collected and used to assess internal consistency (Cronbach’s α) and basic dimensionality checks. For the final analytic dataset, only composite scale scores were retained.

### Eye-tracking data quality and exclusions

Gaze data quality was evaluated based on calibration validity and visual inspection of gaze signal stability during stimulus viewing. Participants were excluded from eye-tracking analyses if calibration validation exceeded the predefined error threshold (≤ 1° of visual angle). No participant met these exclusion criteria; thus, the full sample was retained for fixation-based analyses.

### Quiet-eye (QE) metrics

Quiet Eye was defined following Vickers (1996, 2016):

The final fixation on a task-relevant AOI lasting ≥ 100 ms within the last 500 ms before a decision response. In the present study, Quiet Eye fixation is conceptualized as an observable correlate of attentional allocation during the final decision phase rather than as an index of executive control, self-regulation, or inhibitory capacity.

Two QE variables were extracted:

- QE_reward_ms: Final fixation duration on reward AOI
  Observed range: 185–1,348 ms; M = 752.23, SD = 182.56
- QE_warning_ms: Final fixation duration on warning AOI
  Observed range: 378–1,302 ms; M = 892.92, SD = 174.22.

Fixations were identified using dispersion-based clustering (I-DT algorithm; threshold = 100 ms, 1° dispersion). Only the last fixation before the choice response was considered. Saccades were not classified as fixations and were therefore excluded from QE metrics. AOI assignment was based on the fixation centroid location, and only fixations whose centroid fell within the predefined AOI boundaries were considered task relevant.

### Additional rationale for QE parameter selection

The selected QE parameters follow established conventions in visuocognitive and perceptual–motor research. The duration threshold of ≥100 ms and the 1° dispersion criterion are consistent with standard I-DT fixation algorithms and align with QE protocols used in foundational work by Vickers (1996), Vine et al. (2014), and Wilson et al. (2019). The 500-ms temporal window for defining the final fixation reflects common practice in studies examining the perceptual–cognitive period immediately preceding an action. These thresholds were chosen to maximize comparability with existing QE literature and to ensure consistent detection of the final task-relevant fixation under tightly controlled laboratory conditions.

### Psychosocial risk constructs

Participants completed validated TPB/EPPM-aligned measures using 1–7 Likert-type scales.

Risk Acceptance (4 items, *α* ≈ 0.82).

Example: “I am willing to take health risks if the performance benefits are high.”

Risk Perception (4 items; *α* ≈ 0.80).

Example: “Using strong performance boosters can cause serious long-term harm.”

Attitudes toward Risky Supplements (4 items; *α* ≈ 0.86).

Example: “Using high-stimulant supplements is beneficial for my training success.”

Perceived Behavioral Control (4 items; *α* ≈ 0.83) Example: “It would be easy for me to avoid risky supplements even if others around me use them.”

### Cognitive load manipulation

Before completing the decision task, participants were randomly assigned to:

- Low-load condition (0): simple color-naming task (30 s)
- High-load condition (1): Stroop interference task (incongruent trials; 30 s)

Distribution in our sample:

- 51.2% low-load
- 48.8% high-load

To preserve attentional neutrality immediately prior to the eye-tracking task, no explicit manipulation check was included. This design choice follows established cognitive-load paradigms in rapid decision-making research, where additional checks may introduce priming or visual interference. Consequently, cognitive load is interpreted as an experimentally induced situational condition rather than as an individually quantified subjective state, reflecting transient attentional demands that may shape health-relevant decision processes at the point of choice.

### Task based outcome: risky supplement choice

Participants chose between:

- a safe, moderate supplement option (standard pre-workout), or
- a risky, high-stimulant supplement (ambiguous safety, aggressive claims).

Choice was coded:

- 0 = safe option
- 1 = risky option

Observed rate of risky choice: 36.7%.

Intention (0–100 scale).

After each choice, intention to use the risky supplement was measured:

“How likely would you be to use this product in real life?”

(0 = not at all, 100 = very likely).

Observed: M = 25.51, SD = 20.03.

### Procedure

1. Consent and baseline measures Participants completed psychosocial questionnaires (risk acceptance, risk perception, attitudes, PBC).
2. Cognitive load manipulation Random assignment via automated script.
3. Eye-tracking calibration 9-point calibration; error threshold ≤ 1°.
4. Decision Task

- View single supplement stimulus.
- Eye movements recorded.
- Binary risky choice.
- Intention rating.

#### Mechanistic laboratory design

**Figure 2:**
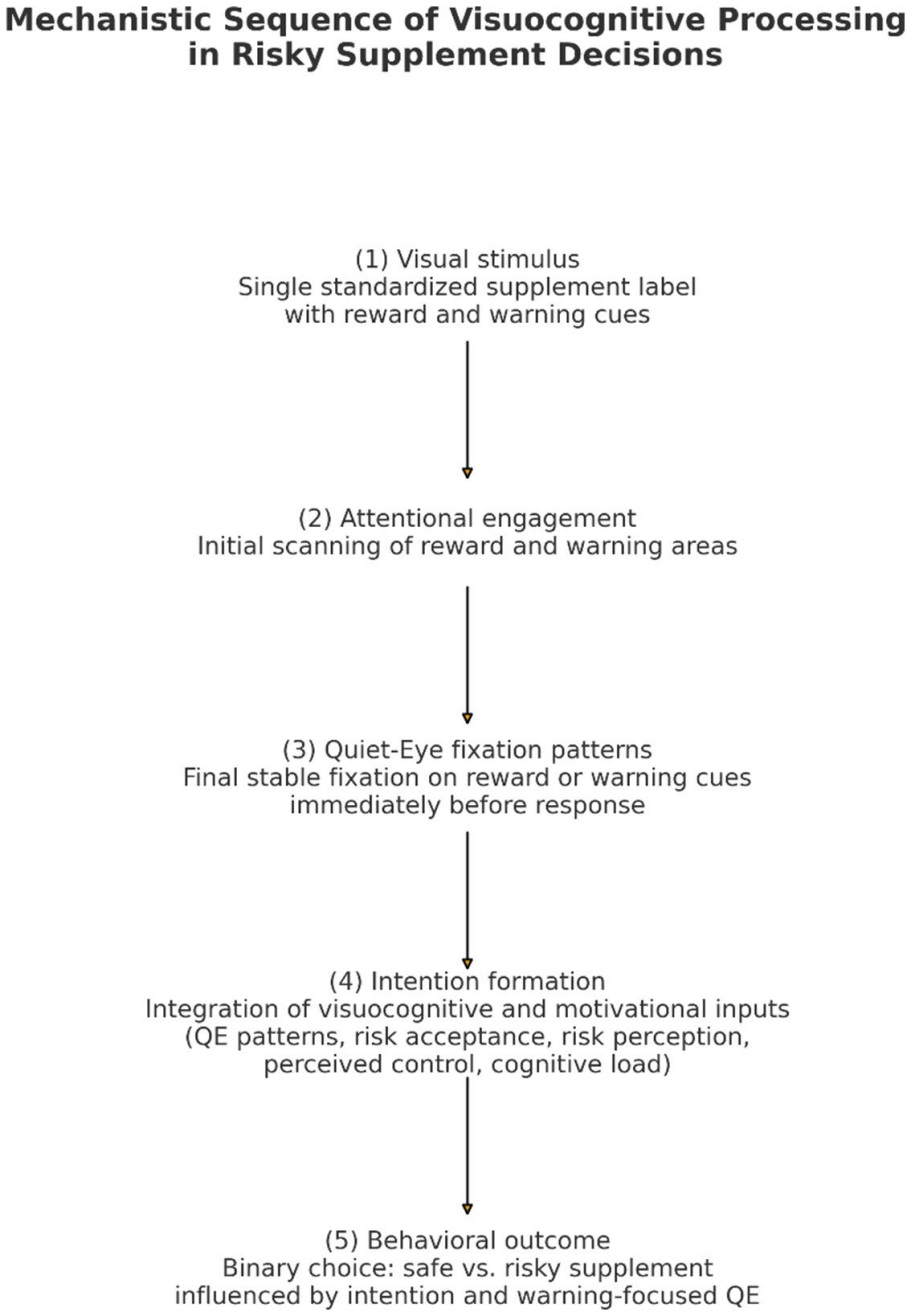
Mechanistic sequence of visuocognitive processing during the decision task. Participants viewed a standardized supplement stimulus, visually engaged with reward and warning cues, exhibited quiet-eye fixation patterns, formed intentions based on visuocognitive and motivational inputs, and made a binary choice between a safe and a risky product. Arrows indicate a conceptual and temporal ordering of processes rather than causal or statistically tested pathways.

This study was designed as a mechanistic laboratory experiment to isolate moment-to-moment visuocognitive patterns under tightly controlled conditions. A single standardized decision trial was used to ensure maximal control over visual complexity, AOI structure, and fixation timing, allowing precise temporal alignment between final fixation patterns and task based choice. Given the single trial paradigm, QE metrics are interpreted as momentary indicators of final attentional allocation rather than stable individual traits; accordingly, we make no reliability claims that would require repeated measures. Consistent with established Quiet Eye research, single-trial or low-trial paradigms can yield valid estimates of attentional stability when the stimulus and temporal structure are fixed and task-relevant (Vickers, 1996; Vine et al., 2014; Wilson et al., 2019). This approach prioritizes internal validity and temporal precision over ecological variability Figure 2.

### Data preparation

- No missing values occurred.
- Continuous predictors were z-standardized prior to regression.
- QE outliers (> 3 SD) were inspected; none required removal.
- Behavioral choice remained binary.

The analytic approach was specified *a priori* and reviewed as part of the study protocol approved by the institutional ethics committee.

### Statistical analysis

Because the decision task consisted of a single standardized trial per participant, all observations were independent at the participant level. Accordingly, all models were specified at the participant level, and no trial-level random effects were required. Analyses were conducted in Python 3.11 (statsmodels).

To enhance statistical transparency and address potential concerns about multicollinearity, we provide the full correlation matrix and reduced predictor models in Supplement S1. These supplemental analyses indicate that the association between QE_warning and the outcome variables remains stable when only core psychosocial variables are included, and that the explained variance in intention aligns with theoretical expectations for TPB-based models.

### Logistic regression predicting behavior

Predictors:

- z(QE_reward), z(QE_warning)
- z(risk acceptance), z(risk perception)
- z(cognitive load)

Outcome: risky choice (0/1) Reporting includes: ORs, 95% CIs, and *p*-values.

Multiple linear regression predicting intention.

Same predictors as above.

Outcome: intention (0–100) Reporting includes: *b*, *t*, *p*, and *R^2^*.

Model validity

- All VIF values < 2.1 (no multicollinearity).
- Residuals showed normal distribution for linear models.
- Logistic model passed goodness-of-fit (Hosmer–Lemeshow *p* > 0.05).

To ensure that the models were not unduly influenced by shared variance among predictors, additional robustness checks were conducted. Models including only QE variables and the central psychosocial constructs (risk acceptance and risk perception) produced virtually identical effect patterns for both task based choice and intention outcomes. This indicates that the predictive contribution of QE_warning is not an artifact of multicollinearity. Consistent with TPB-based research, high correlations between motivational variables (e.g., risk acceptance, intention) are theoretically expected and did not compromise model stability, as reflected in the low VIF values and unchanged coefficient directions across reduced models.

## Results

### Descriptive statistics

Descriptive statistics for all study variables are presented in Table 1. Participants reported moderate levels of risk acceptance (M = 3.80, SD = 1.11) and moderately high levels of risk perception (M = 4.44, SD = 1.06). Mean Quiet Eye fixation durations were longer for warning cues (M = 892.92 ms) than for reward cues (M = 752.23 ms). Mean intention to use the risky supplement was relatively low (M = 25.51, SD = 20.03). Overall, 36.7% of participants selected the risky supplement option.

**Table 1:** Descriptive statistics of key variables.

Variable	Mean	SD	Min	Max
Risk_accept_1_7	3.8	1.11	1.0	6.63
Risk_perc_1_7	4.44	1.06	1.87	7.0
Attitude_iped_1_7	3.25	1.28	1.0	6.32
pbc_1_7	4.07	1.1	1.42	6.88
qe_reward_ms	752.23	182.56	185.0	1348.0
qe_warn_ms	892.92	174.22	378.0	1302.0
Intention	25.51	20.03	0.0	81.7
Risk_choice	0.37	0.48	0.0	1.0
Age	28.19	5.94	18.0	50.0
Training_years	6.37	4.29	0.5	18.5
Load	0.49	0.5	0.0	1.0

All continuous predictors were z-standardized prior to regression analyses.

All continuous predictors were z-standardized before the regression analyses to allow direct comparison of effect sizes.

### Correlations

Bivariate correlations among visuocognitive variables, psychosocial constructs, intention, and task-based choice are shown in Table 2. QE fixation on reward cues was positively correlated with intention (*r* = 0.545) and showed a smaller positive correlation with risky choice (*r* = 0.245). QE fixation on warning cues was negatively correlated with intention (*r* = −0.481) and risky choice (*r* = −0.346). Risk acceptance showed strong positive correlations with both intention (*r* = 0.843) and risky choice (*r* = 0.435), whereas risk perception was inversely correlated with intention (*r* = −0.625) and risky choice (*r* = −0.452). As expected, correlations with intention were generally larger than correlations with task-based choice.

**Table 2:** Correlations among key variables.

	QE reward (ms)	QE warning (ms)	Risk acceptance (1–7)	Risk perception (1–7)	Intention (0–100)	Risky choice (0/1)
qe_reward_ms	1.0	−0.182	0.49	−0.267	0.545	0.245
qe_warn_ms	−0.182	1.0	−0.345	0.112	−0.481	−0.346
Risk_accept_1_7	0.49	−0.345	1.0	−0.49	0.843	0.435
Risk_perc_1_7	−0.267	0.112	−0.49	1.0	−0.625	−0.452
Intention	0.545	−0.481	0.843	−0.625	1.0	0.56
Risk_choice	0.245	−0.346	0.435	−0.452	0.56	1.0

### Logistic regression predicting risky supplement choice

A logistic regression model examined associations between visuocognitive attention, psychosocial variables, cognitive load, and risky supplement choice (0 = safe option, 1 = risky option). The model showed acceptable fit (Hosmer–Lemeshow *p* > 0.05).

Significant predictors

- QE_warning: OR = 0.50, 95% CI [0.32, 0.79], *p* = 0.003
- Risk acceptance: OR = 1.84, 95% CI [1.06, 3.20], *p* = 0.030
- Risk perception: OR = 0.37, 95% CI [0.22, 0.62], *p* < 0.001

Non-significant predictors

- QE_reward (*p* = 0.947)
- Cognitive load (*p* = 0.422)

These results indicate that longer QE fixation on warning cues, lower risk acceptance, and higher risk perception were statistically associated with a lower likelihood of selecting the risky supplement Table 3.

**Table 3:** Logistic regression predicting risky choice.

Predictor	OR	95% CI	*p*
Intercept	0.451	[0.299, 0.681]	0.0
z_qe_reward_ms	0.985	[0.626, 1.548]	0.947
z_qe_warn_ms	0.502	[0.318, 0.791]	0.003
z_risk_accept_1_7	1.844	[1.062, 3.202]	0.03
z_risk_perc_1_7	0.371	[0.222, 0.62]	0.0
z_load	1.177	[0.791, 1.751]	0.422

### Linear regression predicting intention

A multiple linear regression examined associations between visuocognitive attention, psychosocial constructs, cognitive load, and intention to use the risky supplement (0–100 scale). The model explained a large proportion of variance in intention (R^2^ = 0.85). This level of explained variance is consistent with TPB-based intention models in which proximal motivational constructs are theoretically expected to share variance and therefore reflects conceptual saturation rather than statistical overfitting.

Significant predictors

- Risk acceptance: *b* = 11.13, *t* = 13.41, *p* < 0.001
- Risk perception: *b* = −5.57, *t* = −7.74, *p* < 0.001
- QE_reward: *b* = 2.98, *t* = 4.17, *p* < 0.001
- QE_warning: *b* = −4.20, *t* = −6.18, *p* < 0.001
- Cognitive load: *b* = 2.02, *t* = 3.14, *p* = 0.002

Higher intention scores were associated with higher risk acceptance, longer fixation on reward cues, and higher cognitive load, whereas higher risk perception and longer fixation on warning cues were associated with lower intention scores Table 4.

**Table 4:** Linear regression predicting intention.

Predictor	B	*t*	*p*
Intercept	25.511	41.235	0.0
z_qe_reward_ms	2.981	4.166	0.0
z_qe_warn_ms	−4.196	−6.179	0.0
z_risk_accept_1_7	11.129	13.408	0.0
z_risk_perc_1_7	−5.567	−7.745	0.0
z_load	2.017	3.138	0.002

For interpretability, the effect sizes are expressed in descriptive terms. An odds ratio of 0.50 for warning-related QE indicates that a one standard deviation increase in final fixation duration on warning cues is associated with approximately half the odds of choosing the risky supplement. Likewise, the regression coefficient for QE_warning (*b* = −4.20) corresponds to a lower intention score on the 0–100 scale.

### Summary of results

Across analyses, visuocognitive, psychosocial, and situational variables showed distinct patterns of association with intention and task-based choice. Fixation duration on warning cues was consistently associated with both intention and choice, whereas fixation on reward cues was associated with intention but not with behavioral choice once psychosocial variables were included. Psychosocial constructs, particularly risk acceptance and risk perception, showed strong associations with both outcomes. Cognitive load was associated with intention but not with behavioral choice.

For descriptive interpretation, an odds ratio of 0.50 for QE fixation on warning cues indicates that a one standard deviation increase in fixation duration was associated with approximately half the odds of selecting the risky supplement. Similarly, the regression coefficient for QE_warning (*b* = −4.20) corresponds to a lower intention score on the 0–100 scale. All reported effects describe statistical associations within the standardized laboratory task and should not be interpreted as evidence of causal relationships.

## Discussion

The present study examined associations between visuocognitive attention, psychosocial determinants, and cognitive load in risky supplement decision-making among recreational fitness athletes. By integrating Quiet-Eye (QE) fixation metrics with established psychosocial predictors, this work adds a complementary perspective by considering moment-to-moment attentional processes alongside motivational factors. Several findings are discussed with regard to their conceptual and practical relevance. From a health-oriented decision-making perspective, these findings contribute to understanding how visuocognitive attention is allocated during rapid, risk-relevant supplement decisions under cognitive load, a condition characteristic of many fitness environments.

### Associations between quiet-eye fixation on warning cues and risky supplement decisions

A central finding of this study concerns the association between warning-related Quiet-Eye (QE) fixation and risky supplement decisions. Longer final fixations on warning cues were associated with lower intention to use a risky, high-stimulant supplement and with a reduced likelihood of selecting it. These associations remained when established psychosocial predictors, such as risk acceptance and risk perception, were included in the models.

From a visuocognitive perspective, sustained fixation on warning cues may reflect more stable allocation of visual attention to harm-related information immediately prior to choice. This interpretation is consistent with prior sport-cognition research linking longer QE durations to reduced distractibility and more stable task engagement. However, the present findings do not allow conclusions about underlying cognitive control processes. In addition, given the single-trial laboratory paradigm, Quiet-Eye measures reflect momentary attentional allocation during a specific decision episode rather than stable individual differences. Visual engagement with warning information has repeatedly been identified as a prerequisite for effective risk appraisal, as risk-related content can only influence evaluation processes when it is actively attended to rather than merely present (Visschers et al., 2009). This view is consistent with affect-based models of risk evaluation, which emphasize that attentional focus shapes how risk information is experienced and weighted during decision-making, without implying fully deliberative or consciously controlled processing (Slovic et al., 2005).

In contrast, reward-focused QE fixation was associated with higher intention but did not predict task-based choice once psychosocial determinants were considered. This pattern suggests that fixation on reward cues may be more closely related to motivational evaluation, whereas fixation on warning cues is more closely associated with the final decision outcome.

It is important to emphasize that the present findings do not support causal claims regarding the effect of Quiet-Eye fixation on risky supplement decisions. Quiet-Eye metrics are therefore interpreted as visuocognitive markers of attentional allocation during the final phase of decision-making. From a dual-process perspective, visual attention can be understood as influencing how risk and reward-related information is weighted during rapid evaluative processing, without implying that attentional focus alone determines behavior (Kahneman, 2011). This interpretation is consistent with decision-making research using eye-tracking to describe how attentional allocation co-occurs with choice tendencies, without implying that gaze patterns constitute causal determinants of decisions (Orquin and Mueller Loose, 2013). The observed associations are compatible with theoretical accounts proposing that visual engagement with warning cues may facilitate risk appraisal immediately prior to choice, without implying direct causal influence on behavior.

### Psychosocial determinants and visuocognitive attention in risky supplement decisions

Consistent with the Theory of Planned Behavior, risk acceptance was strongly associated with intention and was also associated with a higher likelihood of risky supplement choice, whereas higher risk perception showed the expected inverse associations with both outcomes. These patterns align with extensive prior research demonstrating the central role of motivational and risk-related constructs in supplement and IPED-related decision-making.

Importantly, warning-related Quiet-Eye (QE) fixation showed associations with intention and task-based choice over and above these psychosocial variables. This pattern suggests that visuocognitive attention may capture aspects of the decision process that are not fully accounted for by stable motivational dispositions alone. From an interpretive perspective, psychosocial determinants can be understood as reflecting relatively enduring risk-related orientations, whereas QE-based fixation patterns reflect moment-to-moment allocation of visual attention during the immediate decision phase. This distinction is offered as a conceptual interpretation of the observed associations rather than as evidence for separable causal processes.

The high proportion of explained variance in intention (*R^2^* = 0.85) is consistent with TPB-based models in which intention integrates variance from multiple attitudinal and risk-related components. Comparable levels of explained variance have been reported in previous research on supplement use and related health-risk behaviors. In the present study, this level of explained variance therefore appears to reflect theoretical saturation rather than statistical overfitting.

To address potential concerns regarding multicollinearity, additional robustness checks were conducted using reduced predictor sets, including models that combined QE variables with the two central psychosocial constructs of risk acceptance and risk perception. These reduced models yielded effect patterns that were highly similar to those observed in the full models, indicating that the associations involving QE_warning were not driven by shared variance among psychosocial predictors. As reported in Supplement S1, variance inflation factors were low across all models, further supporting the stability of the reported estimates.

### Cognitive load influences motivation but not task-based choice

Cognitive load exerted a modest but consistent effect on intention but did not translate into task-based choice. From a cognitive load theory perspective, increased task demands primarily affect early evaluative and integrative processing stages, while later decision execution may rely on more automatized or stabilized representations (Sweller, 2016). This dissociation suggests that acute cognitive strain may bias early evaluative processes without overriding more stable attentional and motivational systems during the final decision phase. This pattern is consistent with classic findings in decision research showing that cognitive load can alter preference formation and evaluative judgments without necessarily translating into different choice behavior (Shiv and Fedorikhin, 1999). Similar dissociations between evaluative processing and final choice behavior have been reported in eye-tracking studies of risky decision-making, where attentional dynamics reflect ongoing evaluation without necessarily determining choice outcomes (Fiedler et al., 2012). In real fitness environments characterized by noise, fatigue, and sensory stimulation, load-related fluctuations may similarly influence motivational openness while leaving behavioral restraint intact. This dissociation is consistent with dual-process accounts of decision making, which distinguish between fast, attention and load-sensitive evaluative processes and more controlled processes guiding final action selection (Evans and Stanovich, 2013). Within this framework, cognitive load may bias early motivational evaluations without necessarily determining behavioral outcomes.

### An integrated dual-pathway model

Taken together, the findings can be interpreted as reflecting two complementary pathways involved in risky supplement decision-making.

1. Visuocognitive pathway: stabilized fixation on warning cues was associated with greater visual engagement with harm-related information and with lower intention and behavioral risk.
2. Psychosocial pathway: high risk acceptance and low perceived harm increase intention, which in turn increases risky choice.

These pathways may operate in parallel rather than competing with one another. QE-warning reflects moment-to-moment attentional allocation, whereas psychosocial constructs represent enduring motivational tendencies. This integrated perspective aligns with contemporary decision-neuroscience models emphasizing that attentional gatekeeping shapes how risk and reward signals are weighted prior to action.

Although the dual-pathway interpretation is theoretically grounded and statistically robust, the present study does not establish causal mechanisms for all components of the model. Cognitive load was experimentally manipulated, but QE patterns and psychosocial determinants were observational associations within this controlled setting. Thus, the model should be understood as a mechanistic conceptual account that integrates attentional and motivational pathways, rather than a definitive causal structure. Future experimental and longitudinal designs are needed to test directional assumptions more explicitly.

### Cognitive load, intention, and behavioral choice

Cognitive load showed a modest and consistent association with intention to use the risky supplement but was not associated with behavioral choice. This pattern indicates that increased cognitive demands may be linked to changes in evaluative or motivational processes without necessarily translating into altered choice behavior. In the present laboratory setting, cognitive load appeared to influence intention while leaving the final behavioral decision unaffected.

From an interpretive perspective, this dissociation suggests that acute cognitive strain may be more relevant for early stages of decision evaluation than for the final commitment to action. In fitness environments, which are often characterized by sensory stimulation, fatigue, and competing demands, similar fluctuations in cognitive load may therefore influence motivational openness without overriding more stable attentional or motivational processes that guide behavior. This interpretation remains tentative and should be examined in more ecologically varied settings.

### Integrating visuocognitive and psychosocial perspectives

Taken together, the observed pattern of associations points to a conceptual distinction between visuocognitive attention and psychosocial determinants in risky supplement decision-making. Warning-related Quiet-Eye fixation was associated with both intention and tasked-based choice, whereas psychosocial constructs such as risk acceptance and risk perception were strongly related to intention and, in turn, to task-based choice. Cognitive load, by contrast, was associated with intention only.

Rather than constituting a formal model, these findings are best understood as describing parallel contributions of attentional and motivational factors within the decision process. This perspective is consistent with eye-tracking research in decision science showing that visual attention systematically relates to how information is sampled and weighted during choice, while not being sufficient to explain decision outcomes on its own (Orquin and Mueller Loose, 2013). This interpretation is consistent with decision-making research suggesting that attentional allocation influences how risk and reward-related information is weighted during evaluation, without implying that attention directly determines choice outcomes (Lim et al., 2011; Marteau et al., 2012). Quiet-Eye fixation reflects moment-to-moment allocation of visual attention during the immediate decision phase, whereas psychosocial constructs represent more enduring motivational orientations. Cognitive load appears to modulate intention without consistently influencing choice behavior.

Importantly, the present study does not establish causal pathways linking these components. Although cognitive load was experimentally manipulated, associations involving Quiet-Eye fixation and psychosocial variables were observational within a controlled laboratory context. The integrative interpretation offered here should therefore be regarded as a conceptual framework for organizing the observed associations, rather than as evidence for a definitive causal structure. Future experimental and longitudinal research is needed to examine directional assumptions and potential interactions between attentional, motivational, and situational factors more explicitly.

### Practical implications for prevention

Risky supplements can be situated within a broader continuum ranging from conventional nutritional products to so-called “grey-zone” stimulants and, ultimately, established forms of IPED and AAS use. This continuum perspective is consistent with qualitative research showing that high-stimulant supplements often occupy an ambiguous “grey zone” between conventional nutrition products and illicit IPED use, sharing overlapping motivational and risk-related characteristics (Kimergård and McVeigh, 2014). This continuum, which is well described in the IPED literature, highlights that certain high-stimulant products share motivational and perceptual characteristics with more advanced forms of performance enhancement. While the present study does not address longitudinal transitions along this continuum, the findings may offer insight into early attentional and motivational processes that are relevant at intermediate stages of supplement-related decision-making.

The following practical implications should be interpreted as tentative and hypothesis-generating rather than as direct intervention recommendations.

### Visual design of warning information

This cautious, harm-reduction-oriented framing aligns with recent work emphasizing the complexity of IPED-related harms and the value of contextual, incremental risk reduction approaches rather than one-size-fits-all solutions (Bates et al., 2022). From a prevention and harm-reduction perspective, the results suggest that visual characteristics of risk information may play a role in how supplement-related decisions are evaluated in high-stimulus environments. In this sense, subtle adjustments to visual salience and attentional guidance could potentially support safer decision tendencies without restricting choice autonomy or relying on prohibitive or fear-based messaging.

Importantly, these implications are derived from associations observed under controlled laboratory conditions and do not imply that modifying visual features alone is sufficient to change real-world behavior. Instead, they point to possible leverage points that may complement existing educational and motivational approaches in prevention efforts, particularly in contexts where decisions are made quickly and under substantial sensory stimulation.

### Warning-related information and attentional guidance

Warning-related information is only likely to inform decision-making when it is visually processed rather than merely present. This view is consistent with research on health warning messages showing that the effectiveness of risk information depends strongly on whether warnings attract visual attention rather than merely being present on a product (Hammond, 2011). Evidence from tobacco control research suggests that increased visual salience of warnings is associated with greater noticeability and improved risk-related evaluations, without implying deterministic effects on behavior. The present findings suggest that visual engagement with warning cues may be relevant for how risk information is evaluated at the point of decision. This interpretation is consistent with research on warning design, which emphasizes that warning information can only inform decision processes when it is perceptually salient and visually attended to, without presupposing behavioral compliance (Laughery and Wogalter, 2006). From an applied perspective, this highlights the potential relevance of design features that increase the likelihood that warning information is visually attended to, such as larger font sizes, higher contrast, or placement at eye level in fitness environments. In digital contexts, brief on-screen pauses or structured presentation of warning information may similarly increase the probability that harm-related content is visually registered. These considerations are intended to inform hypothesis-driven future research and design principles rather than to serve as direct recommendations for practice or policy.

### QE-informed attentional approaches

The results also raise the possibility that approaches aimed at stabilizing visual attention on risk-relevant cues could complement existing prevention strategies. For example, brief visual-attention exercises inspired by Quiet-Eye research may help individuals maintain focus on harm-related information in visually dense, high-stimulus environments. Such approaches remain exploratory and should be understood as conceptual extensions of the present findings rather than as evidence-based interventions.

### Risk acceptance as a motivational target

Across analyses, motivational readiness to accept health risks showed strong associations with both intention and behavior. This pattern aligns with motivational research on doping and supplement use indicating that willingness to accept health risks represents a central psychological driver of enhancement-related decision-making, often operating independently of factual risk knowledge (Ntoumanis et al., 2014). This pattern suggests that prevention efforts may benefit from addressing underlying normative beliefs, perceived social expectations, and commonly held assumptions about supplement safety and effectiveness. Focusing on these motivational dimensions may be particularly relevant in contexts where factual risk information alone has limited impact on decision tendencies.

### Timing of risk communication under cognitive load

Because cognitive load was associated with intention but not with behavioral choice, the timing of risk communication may be an important contextual factor. Risk-related information may be processed differently when individuals are fatigued, distracted, or cognitively strained. Accordingly, preventive communication might be more effective when delivered at moments of lower cognitive demand, such as during cooldown phases, post-training conversations, or outside peak exertion periods. This interpretation remains tentative and warrants further empirical examination.

### Summary of practical considerations

Taken together, these considerations suggest that prevention efforts in fitness contexts may benefit from combining motivational approaches with strategies that increase the visual accessibility and attentional processing of risk information. While the present findings are preliminary and derived from a controlled laboratory setting, they point to potentially relevant directions for harm-reduction and early-intervention efforts in environments where supplement-related decisions are made quickly and under substantial sensory stimulation.

### Methodological strengths

The study offers several methodological strengths, including the integration of eye-tracking with validated psychosocial predictors, precise AOI control, preregistered analyses, and robustness checks addressing multicollinearity. The combination of mechanistic visuocognitive metrics with established behavioral frameworks provides a novel and theoretically coherent approach to examining risky supplement decisions.

### Limitations

This study was intentionally designed as a mechanistic laboratory experiment to isolate moment-to-moment visuocognitive processes under maximally controlled conditions. Such mechanistic laboratory approaches are well suited for isolating attentional and decision-related processes that are directly relevant to health-risk evaluation, even when ecological variability is necessarily constrained. While this laboratory-based approach limits direct generalizability to naturalistic supplement-use contexts, it enables precise temporal alignment between visual attention and decision outcomes. Accordingly, the findings should be interpreted as indicators of attentional processes within a constrained task context rather than as direct estimates of real-world supplement-use behavior.

First, the sample consisted of recreational fitness athletes, a population in which high-stimulant and IPED-like supplement use is common. However, the findings may not generalize to higher-risk environments such as competitive bodybuilding or strength-sport subcultures, where social norms, product exposure, and motivational pressures differ substantially.

Second, ecological realism was constrained by the use of a single standardized supplement stimulus and a single-trial decision paradigm. This design ensured strict AOI control and temporal precision but limits conclusions regarding habituation, learning effects, or intraindividual stability of attentional patterns. Future studies should incorporate multi-stimulus and repeated-measures designs to examine how visuocognitive mechanisms operate across more heterogeneous and dynamic decision environments.

Third, although the 60-Hz eye-tracking system used here provides reliable fixation detection under static laboratory conditions, higher-frequency systems may allow more fine-grained analysis of temporal attentional dynamics in future work. In addition, cognitive load was manipulated dichotomously without a manipulation check to avoid interference with the eye-tracking task. While Stroop-based paradigms are well validated, future research should quantify load intensity using subjective or physiological indicators and examine ecologically relevant stressors such as fatigue, noise, or training-induced arousal.

High correlations among psychosocial constructs, particularly between risk acceptance and intention, are characteristic of TPB-based models in which intention integrates variance from proximal motivational antecedents. Robustness checks using reduced predictor models yielded comparable effect patterns, and all variance inflation factors were low, indicating that multicollinearity did not meaningfully compromise model stability. The high explained variance in intention (*R^2^* = 0.85) is therefore interpreted as theoretical saturation rather than statistical overfitting.

Finally, the observed asymmetry whereby reward-focused fixation was associated with intention but not with behavioral choice suggests that attentional engagement with warning cues may play a distinct role during the final decision phase. This interpretation is offered cautiously and requires targeted experimental testing. To support transparency and reproducibility, Supplement S1 reports the full correlation matrix and reduced predictor models, which further corroborate the robustness of the core findings.

### Future research directions

Future research should extend the present mechanistic findings across broader performance and health-risk contexts. First, incorporating multi-trial or repeated-measures designs would allow examination of intraindividual stability, habituation, and learning effects in visuocognitive risk processing. Such designs would also help clarify whether the association between warning-related QE fixation and reduced risk tendencies remains stable, changes, or becomes more efficient with repeated exposure.

Second, more ecologically complex environments, such as strength-training areas, competitive settings, or “hardcore gym” cultures, should be examined to determine how social norms, environmental stimulation, and real-time pressures interact with visuocognitive patterns during supplement-related decisions. Integrating mobile eye-tracking in naturalistic gym settings may help bridge laboratory precision with ecological validity.

Third, combining eye-tracking with neurophysiological markers (e.g., EEG, HRV) may further elucidate how attentional control, arousal, and inhibitory processes jointly contribute to risk appraisal. Such multimethod approaches could help examine whether warning-focused QE fixation is accompanied by markers of conflict monitoring, inhibitory control, or deeper semantic processing of harm-related cues.

Fourth, cluster-analytic approaches could be used to explore stable attentional “risk profiles,” such as predominantly reward-focused versus warning-focused visual patterns. Identifying such profiles may help characterize subgroups of recreational athletes who differ in susceptibility to risky supplements or in their responsiveness to preventive communication.

Fifth, future work may explore QE-informed “risk literacy” approaches that encourage more stable visual attention to harm-relevant information. Evaluating whether such approaches are associated with changes in risk acceptance or improved label comprehension would provide valuable applied insights for harm-reduction programs.

Finally, digital environments, such as fitness apps, supplement marketing feeds, and influencer-driven content, represent high-impact contexts in which visuocognitive mechanisms are likely to play an important role. Examining how body-ideal imagery, social comparison cues, and persuasive product framing guide visual attention could further advance understanding of supplement-related risk behavior in contemporary fitness cultures.

## Conclusion

This study provides initial indications that Quiet-Eye fixation patterns may be associated with risky supplement decisions among recreational fitness athletes. Warning-focused Quiet-Eye fixation was associated with reduced intention and a lower likelihood of risky choice, even when established psychosocial determinants were included in the models. In contrast, reward-focused fixations were linked to motivational tendencies but did not translate into behavioral outcomes, suggesting differentiated roles of attentional allocation and motivational processes in risk-related decision-making. Cognitive load showed a modest association with intention without influencing behavioral choice. Taken together, the findings indicate that visuocognitive attention may represent a complementary component in supplement-related decision processes and may be relevant for understanding how risk information is processed in visually dense fitness environments.

**Figure 3:**
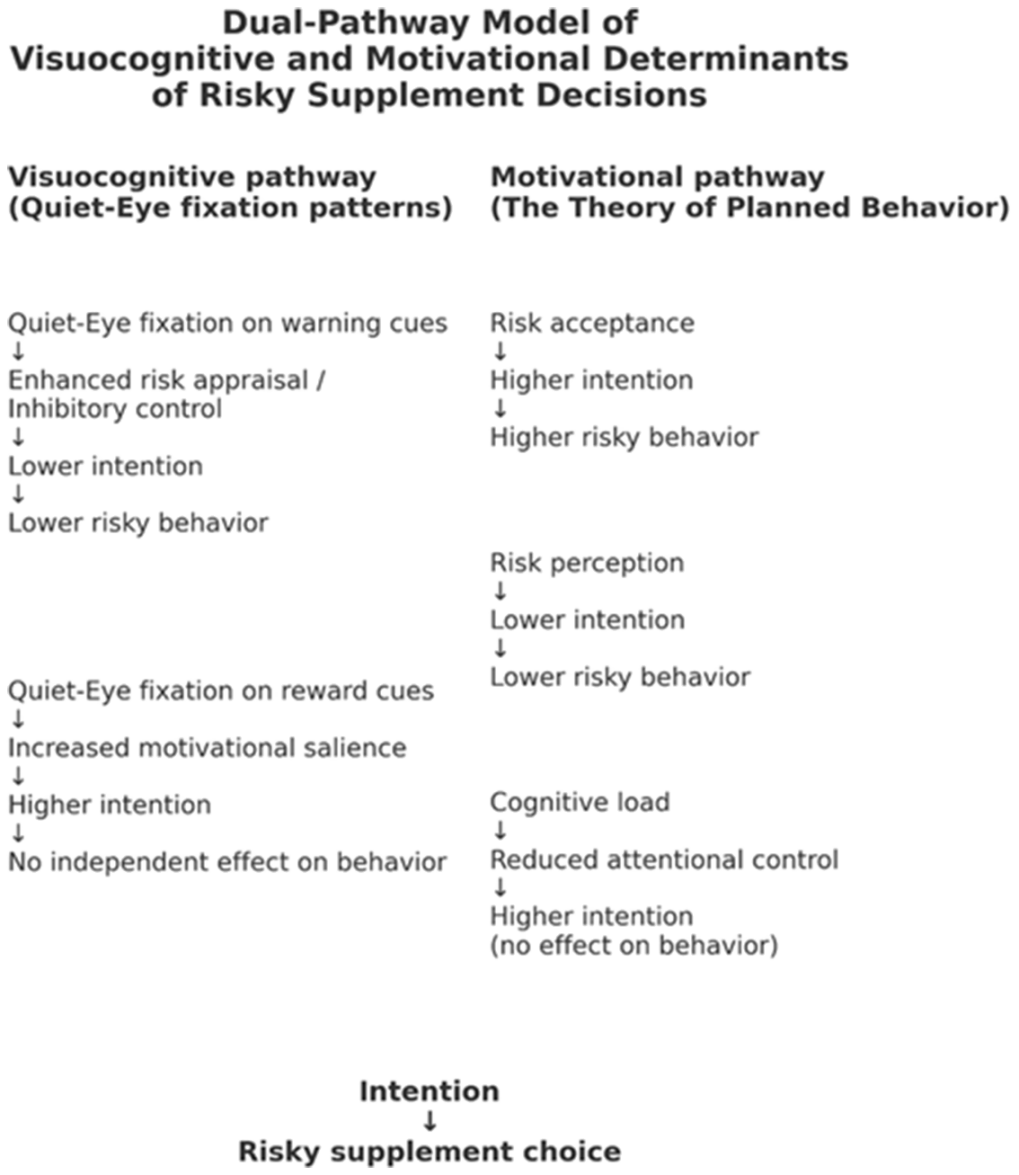
Conceptual dual-pathway framework integrating visuocognitive and motivational determinants of risky supplement decision-making. Warning-focused quiet-eye fixation is associated with lower intention and a lower likelihood of risky task-based choice, whereas reward-focused fixation is associated with higher intention without predicting task-based choice. Motivational determinants derived from the Theory of Planned Behavior (risk acceptance, risk perception) and cognitive load are associated with intention, which functions as the proximal correlate of task-based choice. Arrows represent conceptual and temporal ordering of processes rather than causal or statistically tested pathways.

The procedural sequence of visuocognitive processing is depicted in Figure 3.

## Data Availability

The dataset is not publicly available due to ethical restrictions and participant confidentiality but may be made available by the corresponding author upon reasonable request.
