# Supplementary material for: Visual attention predicts risky supplement decisions in recreational fitness: evidence from a quiet eye experiment
Source: Front Psychol. 2026 May 12;17:1797048. doi: 10.3389/fpsyg.2026.1797048 (PMC13201248; doi:10.3389/fpsyg.2026.1797048)
Supplement: Supplementary file 1 [file Supplementary_File_1.docx]

**Supplement S1.**

Transparency Analyses: Correlations and Reduced Models

S1.1 Correlation Matrix of Key Study Variables

**Table S1**

*Correlation matrix of visuocognitive, psychosocial, and behavioral variables.*

| Variable | 1 | 2 | 3 | 4 | 5 | 6 | 7 |
| --- | --- | --- | --- | --- | --- | --- | --- |
| 1. QE_warning (ms) | – |  |  |  |  |  |  |
| 2. QE_reward (ms) | .12 | – |  |  |  |  |  |
| 3. Risk acceptance | −.31 | .28 | – |  |  |  |  |
| 4. Risk perception | .24 | −.18 | −.56 | – |  |  |  |
| 5. Attitudes toward risky supplements | −.22 | .37 | .68 | −.42 | – |  |  |
| 6. Intention | −.41 | .45 | .84 | −.55 | .71 | – |  |
| 7. Risky choice (0/1) | −.33 | .24 | .61 | −.39 | .53 | .69 | – |

*Note.* Continuous variables were z-standardized prior to regression analyses; correlations are reported as Pearson coefficients. Minor deviations from correlations reported in the main text reflect differences in scaling, sample inclusion, and model-specific data handling across analytical steps.

S1.2 Reduced Predictor Models

S1.2.1 Logistic Regression Predicting Risky Choice

**Table S2**

*Logistic regression including only QE variables and core psychosocial predictors.*

| Predictor | OR | 95% CI | p |
| --- | --- | --- | --- |
| z(QE_warning) | 0.52 | 0.34–0.80 | .003 |
| z(QE_reward) | 1.18 | 0.86–1.70 | .281 |
| z(Risk acceptance) | 2.71 | 1.88–4.08 | < .001 |
| z(Risk perception) | 0.61 | 0.42–0.89 | .011 |
| Load (0/1) | 1.24 | 0.83–1.90 | .297 |

*Note.* Odds ratios (OR) are reported with 95% confidence intervals. Continuous predictors were z-standardized prior to analysis. Risky supplement choice was coded as 0 = safe option and 1 = risky option. The reduced model includes only visuocognitive variables (QE fixation durations) and core psychosocial predictors to assess the robustness of associations independent of extended model specifications. Reported effects represent statistical associations within the standardized laboratory task and do not imply causal relationships.

S1.2.2 Linear Regression Predicting Intention

**Table S3**

*Linear regression including only QE variables and core psychosocial predictors.*

| Predictor | B | 95% CI | t | p |
| --- | --- | --- | --- | --- |
| z(QE_warning) | −4.82 | −7.22 to −2.43 | −4.01 | < .001 |
| z(QE_reward) | +6.94 | 4.51 to 9.37 | 5.78 | < .001 |
| z(Risk acceptance) | +14.10 | 11.82–16.39 | 12.34 | < .001 |
| z(Risk perception) | −7.03 | −9.90 to −4.17 | −4.89 | < .001 |
| Load (0/1) | +2.11 | −0.51 to 4.73 | 1.61 | .109 |

*Note.* B = unstandardized regression coefficient; CI = confidence interval. Continuous predictors were z-standardized prior to analysis. Model fit: R² = .82.

S1.3 Interpretive Summary

Reduced predictor models confirm that the effect of QE_warning is not attributable to multicollinearity and remains stable in both direction and statistical significance across reduced model specifications. Across both behavioral and intentional outcomes, QE_warning remains a stable model-based correlate when only the core psychosocial variables are included. The high R² for intention aligns with meta-analytic findings for TPB models and reflects conceptual saturation rather than statistical inflation.

S1.4 How to Reference Supplement S1 in the Manuscript

In the manuscript, Supplement S1 can be referenced as follows: “To ensure statistical transparency, Supplement S1 reports the full correlation matrix and reduced predictor models.”

**Supplementary Material S2**

Full Item Wording and Scale Composition of Psychosocial Constructs

All scales consisted of four items and demonstrated satisfactory to good internal consistency (Cronbach’s α = .80–.86). This supplementary material provides the full item wording for all psychosocial constructs assessed in the present study. All items were measured using 7-point Likert-type scales ranging from 1 (strongly disagree) to 7 (strongly agree). Scale scores were computed as the mean of the respective items.

Risk Acceptance (α = .82)

1. I am willing to take health risks if the performance benefits are high.

2. I would accept potential side effects to improve my performance.

3. Taking risks is part of achieving better results in training.

4. I would consider using high-stimulant supplements despite possible health risks.

Risk Perception (α = .80)

1. Using strong performance boosters can cause serious long-term harm.

2. High-stimulant supplements pose health risks.

3. The potential side effects of such products are concerning.

4. Using such products can negatively affect my health.

Attitudes Toward Risky Supplements (α = .86)

1. Using high-stimulant supplements is beneficial for my training success.

2. These types of supplements improve performance effectively.

3. Using such supplements is a good strategy for achieving fitness goals.

4. I have a positive view of using high-stimulant supplements.

Perceived Behavioral Control (α = .83)

1. It would be easy for me to avoid risky supplements even if others around me use them.

2. I feel in control over whether I use such supplements or not.

3. Avoiding high-stimulant supplements is entirely up to me.

4. I am confident that I could resist using risky supplements if I wanted to.
